# Veratrum Viride in Hemorrhage from the Gums and Tongue

**Published:** 1860-03

**Authors:** 


*Utility of Veratrum Viride in a case of active hemorrhage from the gums and tongue.*

We find a report of an interesting case of profuse hemorrhage in the January number of the *St. Joseph Journal of Medicine and Surgery*, in which, after the use of various remedial agents, veratrum viride was exhibited in the dose of five drops in a teaspoonful of sweetened water, increasing one drop every fifteen minutes. "The hemorrhage began to cease from the first dose, and about the third dose she appeared fully under the influence of the veratrum, it acting as an emetic, at which time the hemorrhage ceased altogether."

